# Virtual Primary Care for People With Opioid Use Disorder: Scoping Review of Current Strategies, Benefits, and Challenges

**DOI:** 10.2196/54015

**Published:** 2024-12-02

**Authors:** Shawna Narayan, Ellie Gooderham, Sarah Spencer, Rita K McCracken, Lindsay Hedden

**Affiliations:** 1 Department of Family Practice Faculty of Medicine University of British Columbia Vancouver, BC Canada; 2 Faculty of Health Sciences Simon Fraser University Burnaby, BC Canada; 3 Department of Family Medicine Providence Health Care Vancouver, BC Canada

**Keywords:** telehealth, virtual care, primary care, opioid use disorder, opioid agonist therapy, COVID-19, mobile phone

## Abstract

**Background:**

There is a pressing need to understand the implications of the rapid adoption of virtual primary care for people with opioid use disorder. Potential impacts, including disruptions to opiate agonist therapies, and the prospect of improved service accessibility remain underexplored.

**Objective:**

This scoping review synthesized current literature on virtual primary care for people with opioid use disorder with a specific focus on benefits, challenges, and strategies.

**Methods:**

We followed the Joanna Briggs Institute methodological approach for scoping reviews and the PRISMA-ScR (Preferred Reporting Items for Systematic Reviews and Meta-Analyses extension for Scoping Reviews) checklist for reporting our findings. We conducted searches in MEDLINE, Web of Science, CINAHL Complete, and Embase using our developed search strategy with no date restrictions. We incorporated all study types that included the 3 concepts (ie, virtual care, primary care, and people with opioid use disorder). We excluded research on minors, asynchronous virtual modalities, and care not provided in a primary care setting. We used Covidence to screen and extract data, pulling information on study characteristics, health system features, patient outcomes, and challenges and benefits of virtual primary care. We conducted inductive content analysis and calculated descriptive statistics. We appraised the quality of the studies using the Quality Assessment With Diverse Studies tool and categorized the findings using the Consolidated Framework for Implementation Research.

**Results:**

Our search identified 1474 studies. We removed 36.36% (536/1474) of these as duplicates, leaving 938 studies for title and abstract screening. After a double review process, we retained 3% (28/938) of the studies for extraction. Only 14% (4/28) of the studies were conducted before the COVID-19 pandemic, and most (15/28, 54%) used quantitative methodologies. We summarized objectives and results, finding that most studies (18/28, 64%) described virtual primary care delivered via phone rather than video and that many studies (16/28, 57%) reported changes in appointment modality. Through content analysis, we identified that policies and regulations could either facilitate (11/28, 39%) or impede (7/28, 25%) the provision of care virtually. In addition, clinicians’ perceptions of patient stability (5/28, 18%) and the heightened risks associated with virtual care (10/28, 36%) can serve as a barrier to offering virtual services. For people with opioid use disorder, increased health care accessibility was a noteworthy benefit (13/28, 46%) to the adoption of virtual visits, whereas issues regarding access to technology and digital literacy stood out as the most prominent challenge (12/28, 43%).

**Conclusions:**

The available studies highlight the potential for enhancing accessibility and continuous access to care for people with opioid use disorder using virtual modalities. Future research and policies must focus on bridging gaps to ensure that virtual primary care does not exacerbate or entrench health inequities.

## Introduction

### Background

The opioid crisis remains a significant public health challenge [1], highlighted by the rising number of deaths worldwide [2-4]. In North America, illicit drug poisoning deaths can be attributed to a contaminated drug supply saturated with synthetic opioids, particularly fentanyl [5]. Simultaneously, opioid use disorder (OUD) is on the rise [6], with 21.4 million cases worldwide in 2019 [7]. Effective medications for OUD, such as opioid agonist therapies (OATs), reduce illicit drug poisoning deaths [8,9]. Given that primary care services are often at the forefront of diagnosing and treating OUD, evidence shows that accessible primary care services can help achieve better health outcomes for people with OUD [10,11].

The COVID-19 pandemic introduced new barriers to traditional approaches to OUD care, including in-person primary care services, due to the need for social distancing and reduced in-person interactions [12,13]. In response to COVID-19 public health measures, health care systems implemented remote care solutions, transforming the delivery of health care services [14] and accelerating the adoption and use of virtual health care [15-17]. During this same period, much of the progress that had been made to address the opioid crisis was reversed [18-20]; illicit drug poisoning deaths increased as a result of limited health services [21], poisoned supply [22], and solitary drug use [23]. In the context of these challenges, and as a result of pandemic-related public health measures, virtual care emerged as a potential solution to address the unique needs of people with OUD by providing essential health care services remotely [24].

The widespread shift to virtual care facilitated continuous access to care during the pandemic. This shift may have been helpful for individuals with OUD [25,26], many of whom experience barriers to health care due to intersecting effects of criminalization, discrimination, and social marginalization [13]. Virtual care allows clinicians to deliver essential health care services to people with OUD [27,28], offering improved accessibility [29] and the ability to deliver OAT [30] while reducing barriers related to transportation, mobility limitations, and stigma [31]. However, virtual visits may limit clinicians’ ability to fully assess patients’ physical and emotional well-being [27] and may introduce technological barriers for those with limited internet access and low digital literacy [32,33]. In addition, virtual care may not be suitable for all aspects of OUD care as some interventions, such as injectable OAT [34], require regular in-person management [27]. The integration of virtual care into the management of OUD within the primary care context may improve health care delivery, but its limitations, which make in-person visits necessary, must be acknowledged to maximize its efficacy.

### Objectives

Virtual platforms have become increasingly common for primary care services—a trend that is expected to persist beyond the pandemic [35]. Therefore, it is essential to understand the benefits and limitations of current virtual care interventions to shape future health care delivery models and optimize the care provided to patients with social and clinical complexities. This scoping review aimed to synthesize the current knowledge landscape and research gaps and offer insights into the potential of virtual primary care for people with OUD.

## Methods

### Overall Study Design

This review informs an ongoing mixed methods study investigating changes to primary care access and patient outcomes following the rapid introduction of virtual care for people with OUD in British Columbia [36]. We conducted the review in accordance with the Joanna Briggs Institute Reviewer’s Manual [37,38] and the framework suggested by Arksey and O’Malley [39]. We registered our protocol on the Open Science Framework [40] and report our findings using the PRISMA-ScR (Preferred Reporting Items for Systematic Reviews and Meta-Analyses extension for Scoping Reviews) [41] and the PRISMA (Preferred Reporting Items for Systematic Reviews and Meta-Analyses) for Abstracts checklists [42] (Multimedia Appendix 1).

### Eligibility Criteria

We established our eligibility criteria a priori and present these in Textbox 1. We included studies that focused on the concepts of “virtual care,” “primary care,” and “people with OUD” and excluded studies that did not include all 3 concepts. We defined “primary care” as “the provision of integrated, accessible health care services by physicians and their health care teams who are accountable for addressing a large majority of personal health care needs, developing a sustained partnership with patients, and practicing in the context of family and community” [43]. Therefore, primary care settings are those where patients could receive comprehensive care (ie, treatment for any type of health issue) [44], including physician’s offices, community health centers, and specific outpatient clinics. We included some settings that may not traditionally be considered primary care—such as certain opioid treatment programs, syringe service programs, or veterans’ health association programs and clinics—when there was evidence that the setting also attended to health care issues beyond OUD [45-47].

**Eligibility criteria.**

**Inclusion criteria**

- Virtual care modality: synchronous visits, telephone calls, and video calls
- Health service setting: primary care, comprehensive care in physicians’ offices, community health centers, and some outpatient clinics
- Population: people with opioid use disorder with or without comorbidities
- Study type: abstracts, viewpoints, protocols, observational studies, qualitative data, quantitative data, and systematic reviews
- Language: English
- Geographic region: all

**Exclusion criteria**

- Virtual care modality: asynchronous visits, text-message or email-based visits, mobile apps, or unspecified
- Health service setting: psychiatric clinics, substance use disorder clinics, noncomprehensive care provided through opioid treatment programs or syringe services, in-hospital care, prisons, or unspecified
- Population: people aged <18 years and studies in which data specific to opioid use disorder were not distinguished from data for other substance use disorders
- Study type: none
- Language: all other (non-English) languages
- Geographic region: none

### Search Strategy

We developed search strategies [48-52] by combining Medical Subject Heading terms (eg, *analgesics*, *opioids*, *telemedicine*, and *primary health care*) and free-text keywords (eg, *methadone*, *virtual*, and *family doctor*) using Boolean (eg, AND, OR, and NOT), truncation, and wildcard operators. Librarians from the University of British Columbia and Simon Fraser University verified the search strategies. Our final search strategy is included in Multimedia Appendix 2. From December 6, 2022, to December 8, 2022, one reviewer (SN) systematically searched MEDLINE (Ovid) [49], CINAHL Complete (EBSCOhost) [51], Embase (Ovid) [50], and the Web of Science Core Collection [48] with no end date. From May 2023 to June 2023, we searched gray literature using the Canada Commons database [52], Google Scholar, Trip, Grey Matters, and select organization websites (ie, Canada Health Infoway, British Columbia Centre on Substance Use, Centre for Addiction and Mental Health, and Canadian Research Initiative in Substance Misuse). Following the search and extraction, the bibliographies of the included studies were systematically checked for additional references [53].

### Screening

One reviewer (SN) identified and removed all duplicates using Covidence (Veritas Health Innovation) [54], a systematic review software, after which 2 reviewers (SN and EG) screened the identified studies in a 2-stage screening process (title and abstract screening and full-text screening). We achieved a 75% minimum agreement in a pilot test of our screening process and reviewed any conflicts. During screening, some studies did not have well-defined care settings, leaving raters to apply the inclusion criteria differently. Furthermore, the studies spanned a range of methodologies and outcomes, introducing additional challenges in achieving consistent ratings. We reconciled discrepancies at both stages through discussion with the research team and recorded the reasons for study exclusion at each step.

### Data Extraction and Analysis

We (EG, LH, RKM, and SN) created a data extraction chart (Multimedia Appendix 3) to capture relevant information (ie, setting, benefits, challenges, virtual modality, and patient population) in Covidence. To ensure its efficacy, we first piloted this chart on a small sample of studies. On the basis of insights from this pilot test, we refined the chart to better capture the nuances of health service features specific to primary care settings. This revised chart became the cornerstone for the subsequent data extraction from the studies. In parallel, EG and SN conducted quality appraisals of the studies using the Quality Assessment With Diverse Studies (QuADS) tool [55]. The QuADS tool assesses the quality of the methods, evidence, and reporting of studies included in systematic reviews. Our appraisal process involved scoring the studies on a scale from 0 to 3, where the score is derived from an evaluation across several areas, including the clarity of the research aims and robustness of the study design (Multimedia Appendix 4). No study that reached the data extraction stage was excluded due to their QuADS score. Following data extraction and quality appraisal, SN and EG reviewed the data to reach a consensus. We transferred the harmonized data to Microsoft Excel (Microsoft Corp) to conduct inductive content analysis, which led us to identify patterns, which we subsequently organized into themes.

**Figure 1:**
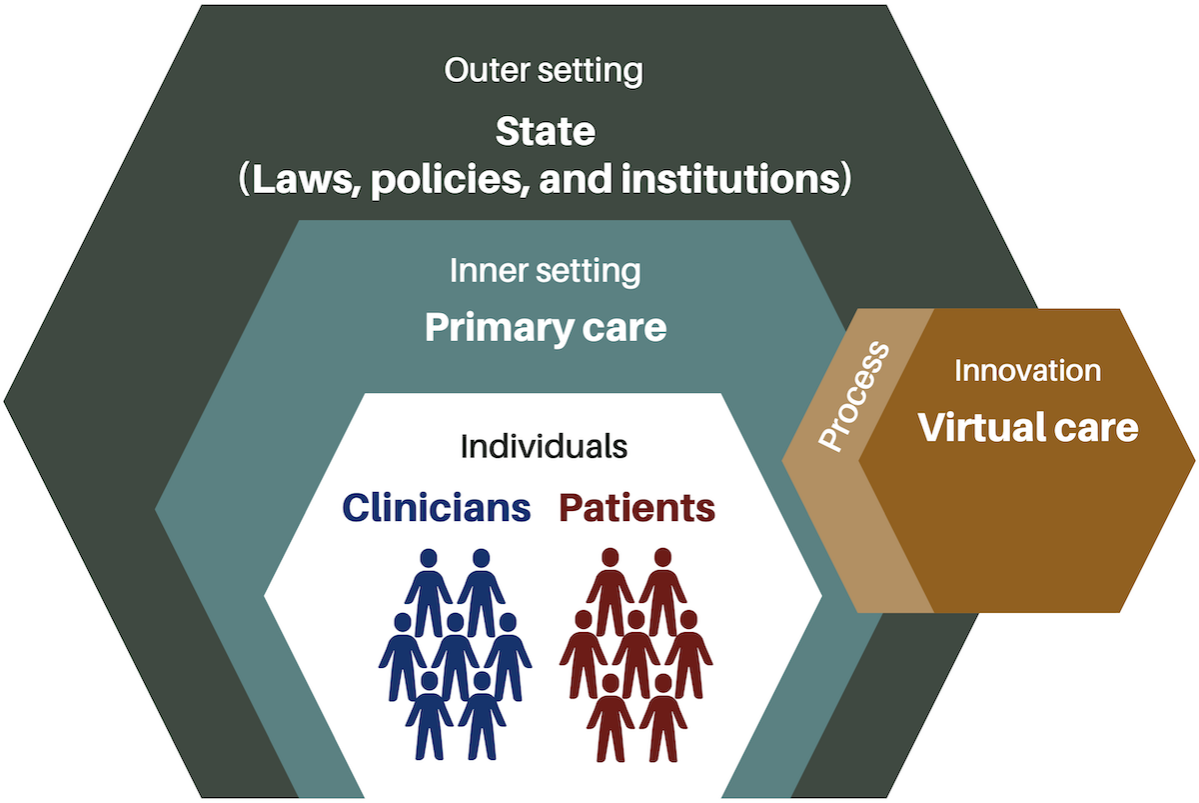
Consolidated Framework for Implementation Research domains as contextualized for this review.

For theme categorization, we used the Consolidated Framework for Implementation Research (CFIR) [56], a widely recognized meta-theoretical model previously applied in other virtual care reviews [57-59]. The CFIR comprises five major domains (Figure 1):

1. Innovation—the change being implemented. In the context of our scoping review, we equate this with synchronous virtual visits in primary care.
2. Outer setting—the context in which the inner setting exists. For this review, the outer setting is the state, including laws, policies, and institutions that inform the provision of and influence access to primary care.
3. Inner setting—the more immediate context where the innovation is applied. While virtual care was implemented throughout the health care system, we were specifically interested in how it was applied and experienced in primary care.
4. Individuals—the actors involved in the provision and experience of the given innovation. Within the primary care milieu, there are 2 principal actors—“innovation receivers” (in this study, people with OUD) and “innovation deliverers” (clinicians offering virtual primary care).
5. Implementation process—the methods and strategies used to implement the innovation. This includes facilitators and barriers that influence the implementation of virtual care.

We discuss facilitators of and barriers to the implementation process (domain 5) within each of the other 4 domains.

## Results

### Overview

**Figure 2:**
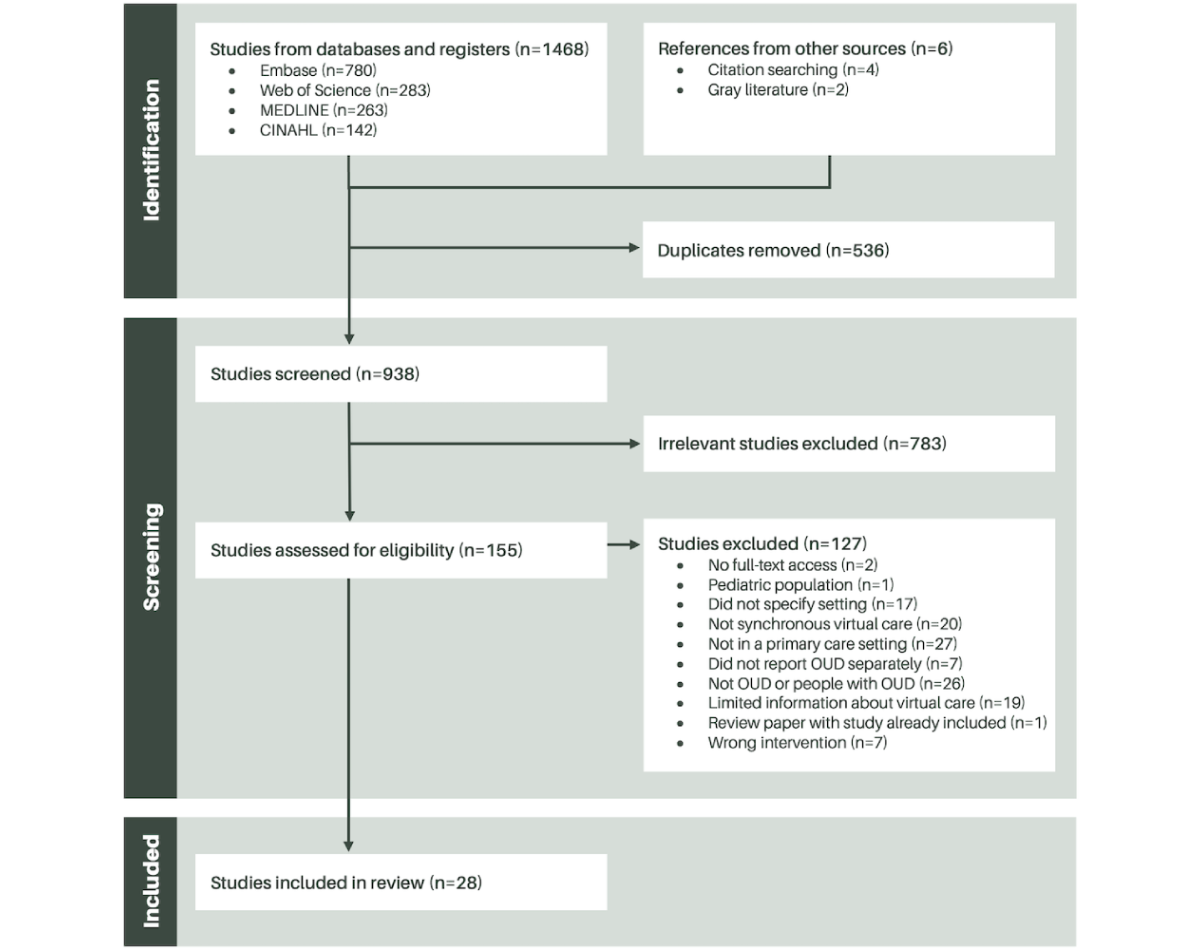
PRISMA (Preferred Reporting Items for Systematic Reviews and Meta-Analyses) flowchart—identification, screening, and inclusion process. OUD: opioid use disorder.

We identified a total of 1474 studies from our searches of the Embase (Ovid; n=780, 52.92%), Web of Science Core Collection (n=283, 19.2%), Ovid MEDLINE (n=263, 17.84%), and CINAHL Complete (EBSCOhost; n=142, 9.63%) databases and through citation searching (n=6, 0.41%). After removing 36.36% (536/1474) of the identified sources due to duplication, we screened the titles and abstracts of 938 studies, retaining 155 (16.5%) for full-text review. After a double review process, we identified 28 papers for data extraction and quality assessment. A PRISMA flow diagram details the exclusions (Figure 2). In this section, numbers within parentheses indicate the frequency and percentage of the studies included in the review that support the statements.

### Study Characteristics

Table 1 provides a summary of the characteristics of each study with its corresponding citation. Most studies (25/28, 89%) were based in the United States and were published after the emergence of COVID-19. Only 14% (4/28) of the studies were conducted before the pandemic [60-63], emphasizing the paucity of research on virtual primary care for people with OUD conducted before 2020. Original research constituted the largest group of studies (11/28, 39%), using various study designs. Most studies had either quantitative (15/28, 54%) or qualitative (4/28, 14%) designs, whereas 4% (1/28) of the studies had mixed methods designs. Other studies reported their findings as commentaries (7/28, 25%) or economic evaluations (1/28, 4%).

The key objectives and results identified in each study are summarized in Table 2. A total of 68% (19/28) of the studies identified incorporating virtual care as part of their primary study purpose or design, whereas the remaining studies (9/28, 32%) included virtual care as an ancillary component of their research. The studies commonly reported the introduction of virtual modalities due to COVID-19 (17/28, 61%), with a few of these papers (6/28, 21%) also examining changes to or increases in existing virtual modality use in response to the pandemic. Outcomes included reports of shifts in health service use, such as changes in appointment modalities (16/28, 57%), frequency or demand (4/28, 14%), and differences in prescription duration (4/28, 14%). We reviewed the number of articles that reported on demographic factors given our interest in equitable practices and understanding disparities in research and health care delivery. Given that it is uncommon for commentaries to report demographics, we excluded them in the following analysis. Of the remaining 22 articles, 11 (50%) reported on sex or gender, 13 (59%) reported on race or ethnicity, and 10 (46%) reported the age of the participants. A total of 18% (4/22) of the studies specified collecting data on participants’ gender [63,64,74,81], whereas 23% (5/22) specified the participants’ sex [36,62,76,77,80]. In total, 9% (2/22) of the studies were unclear on whether they were reporting on sex or gender (eg, *female*/*male* were used, but the study did not specify whether sex or gender were reported) [60,82]. Benefits and challenges of using virtual primary care for people with OUD are grouped within the CFIR domains in the following sections and in Table 3.

**Table 1:** Characteristics of the included studies.

Study	Year	Location	Paper type	Study design
Akoto [61]	2017	United States	Conference abstract	Cohort
Beharie et al [63]	2021	United States	Original research	Qualitative
Behrends et al [64]	2022	United States	Original research	Cross-sectional
Calandra et al [65]	2022	United States	Commentary	Text and opinion
Caton et al [66]	2021	United States	Original research	Cross-sectional
Crowley and Delargy [67]	2020	Republic of Ireland	Brief report	Text and opinion
Griffin et al [68]	2021	United States	Conference abstract	Cross-sectional
Hedden et al [36]	2022	Canada	Protocol	Mixed methods
Hodgkin et al [69]	2021	United States	Review	Economic evaluation
Hser and Mooney [70]	2021	United States	Commentary	Text and opinion
Hser et al [71]	2021	United States	Commentary	Text and opinion
Huskamp et al [72]	2022	United States	Original research	Cross-sectional
Incze et al [73]	2022	Not specified	Conference abstract	Qualitative
Jones et al [74]	2021	United States	Original research	Cross-sectional
Lee et al [60]	2009	United States	Original research	Nonrandomized experimental
Leo et al [75]	2021	United States	Commentary	Text and opinion
Lin et al [62]	2022	United States	Original research	Cohort
O’Gurek [76]	2021	United States	Brief report	Cohort
Patel et al [77]	2022	United States	Brief report	Cohort
Riedel et al [78]	2021	United States	Original research	Cross-sectional
Sahu et al [79]	2022	United States	Conference abstract	Case-control
Sivakumar et al [80]	2022	United States	Original research	Cohort
Snell-Rood et al [81]	2021	United States	Original research	Qualitative
Taylor et al [82]	2021	United States	Conference abstract	Cross-sectional
Thompson et al [83]	2021	United States	Conference abstract	Cross-sectional
Uscher-Pines et al [84]	2020	United States	Original research	Qualitative
Wang et al [85]	2021	United States	Commentary	Text and opinion
Wilson et al [86]	2021	United States	Commentary	Text and opinion

**Table 2:** Objectives, focus of virtual care findings, and results specific to virtual primary care identified during data extraction.

Study	Objective	Virtual care findings	Results
Akoto [61]	Measure adherence to in-person versus video appointments in outpatient primary care	Primary	Of 228 scheduled appointments, 149 (65%) were in person, and 79 (35%) were through video. A total of 93 (60%) in-person and 79 (100%) video appointments were conducted. Virtual care offered benefits such as cost savings and reduced travel time for patients.
Beharie et al [63]	Understand patient perspectives and experiences of care provided by nurse managers	Ancillary	Frequent nurse care manager engagement through phone calls could encourage patient retention. Offering educational and technical assistance for clinicians facilitated virtual care. The Buprenorphine Nurse Care Manager Initiative facilitated patient screening, appointment coordination, pharmacy and medication navigation, provision of care management, and advocacy to enhance treatment engagement and retention. Patients identified barriers such as inflexible schedules for virtual care and unfulfilled promises of phone follow-ups.
Behrends et al [64]	Measure how syringe service programs changed between 2019 and 2020 due to the COVID-19 pandemic	Ancillary	The proportion of syringe service programs offering virtual OAT^a^ prescribing increased from 3% in 2019 to 8% by the end of 2020. Virtual primary care increased from 0% to 8% in the same period. The increase in programs offering virtual mental health care or primary care was statistically significant in facilitating extended virtual service provision. Relaxed prescribing regulations by the DEA^b^ and SAMHSA^c^ facilitated virtual care provision. The rise in virtual care demand may be attributed to disruptions in drug access during the COVID-19 pandemic and increased OAT needs in areas with high fentanyl use.
Calandra et al [65]	Describe how a family medicine center provides care to a clinically and socially complex rural patient population	Ancillary	COVID-19 accelerated the adoption of virtual care to enhance patient accessibility. Virtual care benefits included better antiviral adherence, flexible communication with care coordinators, eased billing for clinicians, reduced travel, fewer work disruptions, enhanced technology access through community agency partnerships, improved attendance, and consistent care. Many clinicians alternated between in-person appointments and telehealth for stable patients with substance use disorder. Challenges included limited broadband access, technology glitches, inadequate technology knowledge, outdated contacts leading to more administrative tasks, mandatory paperwork and laboratory tests to start visits, restricted physical examination scopes, and reduced reimbursement rates compared to in-person visits.
Caton et al [66]	Understand how appointment frequency and type and patient management changed due to the COVID-19 pandemic	Primary	Most (91%) clinics modified care access to either a hybrid of in-person and virtual appointments or all-virtual appointments. Many medical (40%) and mental health care (54%) appointments were virtual. In-person appointments were mostly used for induction of OAT, although some clinics offered this virtually. No clinics required in-person visits for follow-up OAT care. The frequency of follow-up appointments was unchanged. Buprenorphine prescriptions were longer; rates of injectable buprenorphine or naltrexone were unchanged. Larger clinics wrote prescriptions of a longer duration. There was less urine drug screening conducted at most (67%) clinics. Clinics varied in their success with patient retention. Clinicians indicated an increased demand for OAT and mental health care appointments and perceived an increased preference for virtual appointments from patients. Benefits of virtual care included provision of follow-up counseling, medical and behavioral health sessions for stable patients, decreased transportation issues, modified billing for virtual visits, proactive patient outreach, easier medication initiation, and fewer no-shows. However, a challenge to virtual care was clinicians’ reluctance to onboard new patients virtually, perceiving it as high risk and posing a greater liability.
Crowley and Delargy [67]	Describe how to provide virtual OAT care to people with OUD^d^	Primary	COVID-19 transmission risk was reduced. Benefits of virtual care included less travel, easier access for rural patients, enhanced OAT availability, shorter wait times, and the provision of remote psychological support from local drug treatment services, with visit modalities tailored to each patient. A key facilitator of virtual visits was supplying phones to patients in need. However, challenges involved risks from quicker assessments, fewer pretreatment drug screenings, and decreased patient supervision.
Griffin et al [68]	Identify the barriers to virtual care during the COVID-19 pandemic and the feasibility of using community health workers to facilitate virtual care	Primary	A total of 67 community health worker–facilitated virtual appointments were held with 23 unique patients (2.9 mean visits per patient) between April 2020 and December 2020. Approximately half of the appointments were scheduled, and the remaining were walk-in–style virtual appointments. Providing community health workers with smartphones may be a viable strategy to enhance telehealth access and engagement for people experiencing homelessness who might otherwise lack the essential digital tools.
Hedden et al [36]	Understand the impact of expanded virtual primary care on provision of and access to primary care and health outcomes for people with OUD	Primary	The protocol described a mixed methods study with three components focused on virtual primary care for OUD: (1) qualitative interviews with 20 family physicians and 30 people with OUD to understand their experiences with virtual visits, (2) quantitative analysis of population-based administrative data to describe the use of virtual care and its impact on access to services and patient outcomes, and (3) deliberative dialogue sessions to jointly develop resources that promote equitable access to high-quality virtual primary care.
Hodgkin et al [69]	Evaluate the feasibility of office-based treatment	Ancillary	The DEA has allowed buprenorphine to be prescribed virtually, and some insurance companies have temporarily begun covering virtual OAT prescriptions, a service that they previously did not reimburse. Although there are various billing codes, some virtual services receive lower reimbursements than in-person visits, leading to revenue loss for clinics. The continuation of these policies, introduced due to the pandemic to improve OAT access, remains uncertain beyond the COVID-19 pandemic.
Hser and Mooney [70]	Describe high-quality virtual care and opportunities to expand access to virtual care	Primary	Virtual delivery of OAT management was as effective as in-person delivery. Video appointments could be used beyond OAT management, providing opportunities for remote drug screening and mental health care as well. Facilitators of prescribing OAT virtually included more lenient virtual regulations. The collaboration between primary care and seasoned virtual clinicians allowed for more comprehensive access. Factors driving virtual referrals included a shortage of X-waivered clinicians, an absence of behavioral health services, patients’ logistical issues, and managing clinically complex cases. Virtual care offered benefits such as eliminating travel, crucial for rural areas, and provided clinic flexibility and broader patient access. Several telemedicine firms had infrastructure in place, with 24/7 services from licensed clinicians and diverse payment options spanning many areas in the United States. This addressed challenges in rural areas, better catering to patient preferences regarding stigma and privacy. However, rural communities faced challenges such as limited economies of scale, dependency on public payers, and low patient numbers. Many rural residents lacked proper internet and smartphone access. Moreover, some patients were skeptical of digital health care quality. Regulations posed challenges to clinicians in terms of licensing and reimbursement, whereas patients struggled with technology use.
Hser et al [71]	Describe the implementation and effectiveness of virtual care in rural settings	Primary	Clinics provided virtual care (before the COVID-19 pandemic) to address capacity limitations, improve access to mental health care for complex or nonadherent patients, provide more options for patients, and reduce waitlist times. In clinics offering virtual appointments to all patients, most people with OUD did not accept a referral to the virtual program. Rural health centers could expand their capacity and improve patient care by collaborating with established telemedicine vendors. Through a structured protocol, they defined services, referral methods, and communication strategies to address patient retention. To facilitate this, clinics provided spaces and devices for patients, initiated community outreach, and offered additional training for clinicians. However, challenges remained. Rural areas often lacked adequate internet and device access. There were initial setup delays, insurance inconsistencies, regulatory hurdles, and uncertainty about virtual care aligning with treatment philosophies. Some patients also expressed hesitancy toward unfamiliar web-based platforms and the absence of private spaces for virtual visits, whereas clinics grappled with managing patient relationships remotely.
Huskamp et al [72]	Understand clinician use of virtual modalities and comfort with initiating OAT remotely	Primary	Note: the results include primary care physicians (40%), psychiatrists, NPs^e^, and physician assistants. Of 602 clinicians, 58% did not use virtual care for their patients with OUD in 2019. Of the 506 clinicians who provided OAT initiation in the previous month, 60% used virtual modalities, which accounted for 34% of all of their initiation visits. The remaining 40% of OAT-initiating clinicians only provided in-person appointments. In total, 56% of clinicians used virtual modalities for all OUD appointments. Of the 303 clinicians who provided at least one virtual initiation in the previous month, 33% used video for most of their initiation visits versus 8% who used predominately phone. Among this same subset of clinicians who offered virtual initiation in the previous month, 42% were primary care physicians. Facilitators of virtual care included relaxed OAT regulations, experienced clinicians, use of home urine screening kits, remote patient monitoring, Veterans Administration EMR^f^ guidance, clinician support initiatives, and virtual care training during residency. Challenges included clinician hesitancy toward virtual OAT initiations, liability concerns, safety issues, perceptions of diminished care quality with phone visits, constraints previously imposed by the Ryan Haight Act, clinician discomfort, and the “digital divide” that limits access to video visit technologies for many vulnerable patients.
Incze et al [73]	Describe patient experiences of receiving OUD care in primary care	Ancillary	Respectful, team-based care facilitated flexibility for virtual appointments. Virtual care for people with OUD offered benefits such as flexibility in scheduling appointments. Furthermore, it reduced feelings of discrimination and stigma. Participants felt that, when accessing virtual care for substance use disorder, it was akin to consulting a physician for any other medical concern.
Jones et al [74]	Assess adaptations to care for people with OUD, including the use of virtual care, by clinicians during the COVID-19 pandemic	Primary	Before the COVID-19 pandemic, 21% of clinicians prescribed OAT virtually for their established patients. Since the COVID-19 pandemic, clinicians reported either no change (60%) or increased demand (29%) for treatment from patients. In total, 33% of clinicians prescribed virtually to new patients, among whom 87% reported no difficulties with virtual buprenorphine induction. Where clinicians did encounter problems with virtual induction, these included withdrawal symptoms (52%), oversedation (8%), allergic reaction (2%), or other challenges (41%). Clinicians used several virtual modalities with new patients, including computer with video (54%), phone (54%), phone with video (46%), tablet with video (19%), and others (3%). For existing patients, virtual modalities included phone (64%), computer with video (51%), phone with video (47%), tablet with video (21%), and others (8%). The most common applications used for virtual appointments were Zoom (36%), FaceTime (15%), and Skype (6%). Clinician strategies for virtually monitoring patients included prescribing naloxone (50%), mental health care with video (51%) and without video (47%), group counseling with video (12%) and without video (6%), and pill and film checks with video (9%). Since the COVID-19 pandemic, most (72%) clinicians did not change the length of buprenorphine prescriptions, whereas some (24%) increased the number of days. Most clinicians (73%) did not change their use of long-lasting or extended buprenorphine, whereas for others (18%), it was not available. Virtual care was particularly embraced by younger clinicians, many of whom had previous experience with virtual prescribing and handled a large number of OAT patients. These services could be found across various practice settings, but there were notable challenges such as initiating buprenorphine treatments virtually and managing withdrawal symptoms from a distance. Technological barriers such as unreliable systems for both patients and clinicians posed significant problems, compounded by concerns about security and privacy. Financial challenges related to accessing technology and inadequate reimbursements for virtual services further complicated the situation. Some physicians preferred in-person consultations, and some clinics continued to operate primarily in person. Additional barriers included physician hesitation to treat remotely without a previous in-person interaction and specific challenges tied to home inductions. The lack of awareness of DEA exemptions was also a hindrance. Clinicians were less likely to prescribe buprenorphine virtually to new patients without an in-person examination if they were male (OR^g^ 0.83) compared to female, practiced in the Midwest (OR 0.74) or the South (OR 0.68) as opposed to the Northeast, worked in suburban (OR 0.80) or rural areas (OR 0.67) rather than urban settings, were physician assistants (OR 0.52) compared to physicians, or worked in an emergency department (OR 0.52) rather than in an office-based solo practice.
Lee et al [60]	Describe the outcomes of a home-only OAT induction program for patients accessing office-based primary care OUD treatment	Ancillary	Of the 103 participants who received home induction for buprenorphine between August 2006 and January 2008, a total of 73% returned for their scheduled week 1 visit, 17% did not return but did communicate with staff, and 11% had no contact with the clinic following induction. There were no reports of issues with sublingual dosing, no participants reported severe withdrawal or adverse events, and few (5%) reported buprenorphine-prompted withdrawal symptoms. In a convenience subsample of 17 patients, 18% contacted staff as instructed on induction days 1-3, a total of 42% contacted staff on days 4-6, a total of 24% did not contact staff but did return messages, and 35% did not respond to follow-up. These postinduction support calls lasted an average of 3-4 minutes and focused on the induction experience. These calls were considered infrequent due to low need (attributed to the patient education pamphlets provided), low rates of complications, and patients’ previous experience with buprenorphine.
Leo et al [75]	Describe changes in access to care for people experiencing homelessness resulting from a community partnership that used virtual care during the COVID-19 pandemic	Primary	Working with existing frontline organizations to provide mobile health van deliveries of buprenorphine to patients following a virtual care appointment ensured continued access to medication. Benefits of the program included weekly multidisciplinary meetings to ensure patient follow-up, medication delivery via a mobile health van, and prioritizing team members who had long-standing therapeutic relationships with patients. In addition, the mobile clinic was equipped with phones for virtual visits and, due to DEA regulatory changes, mobile health organizations could prescribe buprenorphine via video or phone. Barriers experienced were related to generational distrust toward health care systems among racialized communities.
Lin et al [62]	Understand how virtual care use changed over time, which patients are using virtual care, and any differences in health outcomes between in-person and virtual care	Primary	Virtual prescribing of buprenorphine increased 3.5 times between 2012 and 2019, from 2% to 8% of patients receiving buprenorphine. Individual mental health care for OAT patients also increased, whereas group mental health care did not. Primary care only accounted for 0.3% of clinic settings in which virtual OAT management was provided. Patients receiving virtual buprenorphine management were more likely to have depressive or anxiety disorders but less likely to have psychotic, alcohol use, stimulant use, or cannabis use disorders than patients receiving buprenorphine management in-person. Health service use was also higher for patients receiving buprenorphine management virtually (a median of 39 appointments, 4 of which were virtual) than for those receiving buprenorphine management in-person (a total of 20 appointments). Patients accessing their OAT management virtually also had a higher median number of days on their buprenorphine prescription (722) than in-person patients (295). Patients receiving buprenorphine management virtually accessed an average of 18 mental health-related visits (1 of which was provided virtually) compared to 15 mental health-related visits for individuals receiving buprenorphine management in-person. Benefits of virtual care included enhanced access to care in rural areas, increased treatment retention, greater accessibility overall, and regulatory changes that favored telehealth. Especially during the COVID-19 pandemic, with barriers to in-person treatment prevalent, virtual care may have improved engagement and use of buprenorphine. Facilitators of virtual care included the introduction of the DEA waiver to prescribe virtually and in community-based settings. Younger, female, and White patients were more likely to use virtual care. Conversely, challenges involved the necessity for patients to be more stable for this approach. Black patients were less inclined to receive their buprenorphine management virtually.
O’Gurek [76]	Understand how appointment adherence is affected by a disaster management plan and virtual care protocols	Primary	Following a change to virtual modalities (video or phone based on patient preference), virtual appointment adherence rates from March 16, 2020, to April 30, 2020, were 92% compared to 74% adherence to in-person appointments between January 1, 2020, and March 13, 2020. There was a statistically significant difference between no-show rates in the pre–COVID-19 (26%) and COVID-19 (8%) periods studied. Benefits of virtual care included cost savings for patients, efficient use of clinician time evidenced by lower no-show rates, and reduced expenses on office space and staff. Virtual care also enhanced access to services. It was also convenient and decreased the need for travel. Guidelines for ongoing care during the pandemic supported its implementation, and the introduction of web-based recovery resources to support behavioral health therapy provided comprehensive recovery care. Conversely, barriers involved initial costs for HIPAA^h^-compliant technologies, restricted broadband access especially in rural regions, and challenges integrating virtual health services that comply with Title 42 (Part 2) of the Code of Federal Regulations–Confidentiality of Substance Use Disorder Patient Records.
Patel et al [77]	Describe the characteristics of patients and clinicians who used virtual care during the COVID-19 pandemic	Primary	Between March 13, 2020, and May 30, 2021, a total of 15% of inductions were provided virtually. The highest use of virtual inductions (34%) was in April 2020, and this dropped to 14% in May 2021. During this time, primary care physicians and NPs conducted 31% and 16% of virtual inductions, respectively; a psychiatrist was more likely to conduct a virtual induction (OR 2.01) than a primary care clinician. Facilitators for virtual care adoption included the Ryan Haight Act waiver, community broadband accessibility, community programs educating patients on virtual visit procedures, and financial assistance for practices to cover initial virtual care setup costs. Larger practice settings facilitated the implementation of virtual care. Conversely, barriers involved the “digital divide,” where a lack of reliable technology tools and internet access impeded virtual care use for marginalized populations, potentially widening care disparities. Solo practitioners or primary care clinicians may not have the necessary resources to offer OUD care using virtual modalities. Furthermore, equity-deserving groups, including Black patients, rural residents, and those with lower income, were less likely to experience virtual OAT induction. Compared to solo practitioners, clinicians in larger practices were more likely to use virtual care for OAT induction.
Riedel et al [78]	Understand clinician perspectives on virtual care during the COVID-19 pandemic, including the frequency, barriers to, effectiveness of, comfort level with, and compensation of virtual appointments	Primary	In 2019, most clinicians (58%) did not use virtual care for people with OUD. Clinicians reported in the fall of 2020 that, in the previous month, they had conducted most (57%) of their appointments virtually, both using video (37%) and audio-only (20%) modalities. Primary care clinicians conducted 50% of their appointments in-person, whereas they used video (31%) and audio-only (20%) modalities for virtual appointments. NPs and certified nurse specialists saw patients virtually using video (38%) and audio-only (20%) modalities. Physician assistants saw patients virtually using video (30%) and audio-only (14%) modalities. If reimbursement rates did not change, most (80%) of the clinicians said that they would continue with virtual care. If reimbursement rates were 25% lower for virtual than for in-person appointments, only some (40%) said that they would offer virtual care. Facilitators of virtual care included the Ryan Haight Act waiver and clinician comfort using video modalities with clinically stable patients. Furthermore, clinicians with more patients with OUD used virtual appointments and felt more comfortable using video with less stable patients. Certain practices, especially solo and unspecified “other” types, leaned into using video, and a substantial portion of practitioners (63%) affirmed the efficacy of virtual visits compared to in-person visits. Conversely, barriers included preference for audio-only visits due to technological barriers and a lack of patient readiness or resources. Challenges also emerged at the clinician level, with technological and infrastructural issues (eg, lack of HIPAA-compliant technology), licensing regulations, and a perception among some (33%) that virtual care was less effective than in-person care. In addition, after the pandemic, a significant number of clinicians (70%) expressed a preference for returning to in-person visits, revealing a nuanced landscape of clinician perspectives on the sustained application of virtual care in OUD management. Approximately 33% of respondents believed that licensing regulations restricted them from using virtual care for people with OUD. Approximately 49% were comfortable with video consultations for patients who are less stable. Only 3% did not want any virtual visits, whereas 28% wanted them in special situations, such as for patients with mobility challenges or those who were traveling. Meanwhile, 39% favored virtual care often but solely for their existing patients, and another 29% were amenable to using it regularly for both new and established patients.
Sahu et al [79]	Evaluate how virtual care affected patient continuity for individuals receiving buprenorphine	Primary	Among patients seen virtually, most (61%) received follow-up care, which was higher than the rate for patients seen in person (49%). Most patients (80%) experienced at least one socially-determined barrier to their health (eg, access to housing and transportation). Benefits included improved continuity and accessibility of care. Telehealth could also mitigate challenges such as transportation, work commitments, childcare, and other demands, making care more attainable.
Sivakumar et al [80]	Assess whether a differentiated care model for combined HCV^i^ care and OUD screening that used virtual modalities reduced clinical demands during the COVID-19 pandemic	Primary	Combining HCV and OUD care can be successful, providing timely treatment, whether via phone call, video call (for buprenorphine), or in-person (for methadone). Of those patients with HCV and OUD, 45% were initiated on buprenorphine, and 19.4% were initiated on methadone as well as direct-acting antivirals in addition to those already receiving OAT at baseline. In total, 84% of those with HCV and OUD received treatment for both conditions. Telehealth was beneficial for individuals with unstable access to regular in-person care due to socioeconomic or structural issues such as housing instability, lack of insurance, or stigma from health care settings. Limited clinician oversight was required for virtual OAT prescriptions, and in-person contact was not mandatory for buprenorphine prescriptions. Clinically stable patients could opt for take-home dosing with methadone, and phone communication proved sufficient for patient engagement. However, there were concerns about medication adherence. Regulations still required in-person induction for methadone treatment during the pandemic. In many settings, virtual care reimbursement required broadband internet and videoconferencing. To promote health equity, a human rights approach over a reimbursement-driven approach must be prioritized wherein standard phones rather than smartphones can be used for virtual care.
Snell-Rood et al [81]	Capture clinician perspectives on the facilitators of and barriers to rural care	Ancillary	Virtual care was not sufficiently available despite efforts for its integration. The use of virtual modalities, which allows some care providers to work remotely, can impede relationship building between care providers and with patients; this can result in medication errors due to unfamiliarity with patients and insufficient chart reviews. The loosened telehealth regulations may not have sufficiently aided rural organizations, which could require support in establishing lasting connections with remote clinicians. Rural residents often lacked essential broadband and technology for telehealth. Virtual care was often described by participants as an option that their organizations were exploring rather than implementing. Furthermore, virtual care implementation within organizations fluctuated due to staff turnover or equipment changes.
Taylor et al [82]	Capture patient perceptions of a new program offering primary care–based Suboxone induction during the COVID-19 pandemic	Ancillary	A total of 76% of the participants reported being able to call in for an appointment. Benefits of virtual care included lower COVID-19 exposure risk.
Thompson et al [83]	Understand the barriers to care provision associated with OAT	Ancillary	A total of 70% of primary care clinicians with a buprenorphine waiver used virtual appointments for people with OUD. A significant benefit was that most waivered clinicians were using virtual care to support patients in rural areas, filling gaps caused by the absence of OUD services in those regions.
Uscher-Pines et al [84]	Describe how virtual and in-person care were used during the COVID-19 pandemic, as well as the barriers and quality-of-care implications	Primary	As of April 2020, almost all clinicians (94%) used virtual care for people with OUD, and most (72%) did not offer any in-person visits. Most clinicians (83%) offered either phone or video appointments. While most clinicians indicated a preference for video over phone appointments, a median of 20% of appointments were conducted via phone. Common video platforms included Zoom, FaceTime, and Google Meet. Virtual appointments took place in patients’ homes or outside the clinic following an in-person urine toxicology screening, although the frequency of these tests decreased or ceased. For some clinicians, appointment frequency decreased, and the length of prescriptions increased. Overall, clinicians felt that transitioning to virtual care went well and that it would be retained in the future in conjunction with in-person care. Benefits of virtual care included enhanced patient access and convenience as it eliminated the need for travel and reduced no-show rates. Patients had flexible options for care outside typical business hours, which decreased wait times and aided in more efficient clinic operations. Virtual care also alleviated pandemic-induced anxiety and provided clinicians with a unique insight into patients’ environments, fostering a deeper connection. Adjustments due to COVID-19, such as reduced urine screening and an increased supply of OAT, were received positively. Many found virtual visits comparable to in-person visits and anticipated their continued use after the pandemic for their convenience. However, challenges persisted, including a perceived lack of structure and accountability, technological barriers, shorter visit durations, and physical requirements such as laboratory tests. In addition, there was hesitation in onboarding new patients remotely, especially those with recent relapse. The future of virtual care remained uncertain given potential changes to reimbursement and regulations.
Wang et al [85]	Describe how virtual care was used during the COVID-19 pandemic	Primary	Examples from the clinics highlighted how virtual appointments facilitated a low-barrier, faster, and more flexible OAT induction and access to naloxone. Virtual care for OUD reduced gaps in treatment and mortality. Regulatory modifications streamlined the initiation process for patients seeking buprenorphine as in-person evaluations were not mandatory. This low-barrier approach, aided by the remote guidance of medical students and patient navigators, enabled patients to conveniently access virtual care. Phones with comprehensive plans were distributed to those in need, ensuring that community health workers could facilitate virtual care during outreach activities. This augmented approach enhanced access to OUD treatment, breaking down barriers ranging from limited clinician availability; racial disparities; and other patient challenges such as stigma, transportation issues, and competing priorities such as housing and food. However, in-person interactions were still frequently viewed as more engaging and motivational, with additional benefits for clinicians, such as urine toxicology tests. Virtual care also introduced new disparities as rural residents, racial minority groups, older adults, and low-income individuals may lack the requisite technological equipment or digital literacy. Connectivity was a persistent issue, especially in rural areas. The absence of reliable smartphone access among older patients, those with limited English proficiency, or those of a lower socioeconomic background further exacerbated this challenge. Clinicians sought evidence-backed virtual care protocols to better understand patient management in this digital era amid concerns that virtual care may compromise the treatment structure or increase medication diversion.
Wilson et al [86]	Describe how virtual care was used during the COVID-19 pandemic to provide patient-centered care	Primary	This clinic took a harm reduction approach during the COVID-19 pandemic by spacing out OAT appointments when appropriate, electronically prescribing “buprenorphine/naloxone,” and offering phone appointments when possible and based on patient needs. Clinicians also ensured that all patients had an active prescription for naloxone. They noted that virtual care reduced the risk of COVID-19 exposure, which has been supported by insurance changes to reimbursement for such visits. Guidance from the DEA on electronic prescribing, along with a centralized hub for resources on billing, regulations, and compliance, further facilitated virtual care adoption. Virtual care was particularly beneficial for low- to moderate-acuity patients who did not require in-office visits or urine drug screens, offering added appointment flexibility. Conversely, inconsistent cell phone service and limited broadband internet could impede virtual care access. Insurance reimbursement could pose difficulties, and there were concerns that the absence of urine screenings might lead to relapse among some patients. Some clinicians deemed virtual care unsuitable for high-acuity patients such as recent drug users, pregnant women in their third trimester, or those new to care, necessitating in-person appointments.

^a^OAT: opioid agonist therapy.

^b^DEA: Drug Enforcement Administration.

^c^SAMHSA: Substance Abuse and Mental Health Services Administration.

^d^OUD: opioid use disorder.

^e^NP: nurse practitioner.

^f^EMR: electronic medical record.

^g^OR: odds ratio.

^h^HIPAA: Health Insurance Portability and Accountability Act.

^i^HCV: hepatitis C virus.

**Table 3:** Benefits and challenges of virtual primary care for people with opioid use disorder organized by Consolidated Framework for Implementation Research (CFIR) domains and constructs (N=28).

CFIR domain				CFIR construct		Studies, n (%)		References
**Innovation: virtual primary care**								
	**Benefits**							
		Modality options	Innovation and adaptability		7 (25)		Sivakumar et al [80] Uscher-Pines et al [84] Huskamp et al [72] Jones et al [74] Hser et al [71] Wilson et al [86] Crowley and Delargy [67]	
	**Challenges**							
		—^a^	—		—		—	
**Outer setting: state**								
	**Benefits**							
		Regulation changes	Policies and laws		11 (39)		Lin et al [62] Behrends et al [64] Patel et al [77] Huskamp et al [72] Hser and Mooney [70] Jones et al [74] Wang et al [85] Riedel et al [78] Wilson et al [86] Hodgkin et al [69] Leo et al [75]	
		COVID-19 public health measures	Critical incidents		4 (14)		Lin et al [62] Uscher-Pines et al [84] Wilson et al [86] Taylor et al [82]	
		Billing changes	Policies and laws		4 (14)		Calandra et al [65] Hser and Mooney [70] Caton et al [66] Hodgkin et al [69]	
		Insurance reimbursement	Policies and laws		2 (7)		Wilson et al [86] Hodgkin et al [69]	
		Support for up-front costs	Financing		1 (4)		Patel et al [77]	
		Community engagement	Partnerships and connections		1 (4)		Hser et al [71]	
		Changes in the drug market during the pandemic	Critical incidents		1 (4)		Behrends et al [64]	
	**Challenges**							
		Regulations	Policies and laws		7 (25)		Sivakumar et al [80] Huskamp et al [72] Hser and Mooney [70] O’Gurek [76] Jones et al [74] Hser et al [71] Riedel et al [78]	
		Billing	Policies and laws		4 (14)		Beharie et al [63] Sivakumar et al [80] Calandra et al [65] Jones et al [74]	
		Insurance reimbursement	Policies and laws		4 (14)		Hser et al [71] Riedel et al [78] Wilson et al [86] Hodgkin et al [69]	
**Inner setting: primary care**								
	**Benefits**							
		Clinical resources available	Available resources		6 (21)		Huskamp et al [72] Hser and Mooney [70] O’Gurek [76] Wang et al [85] Hser et al [71] Wilson et al [86]	
		Technological supports	Available resources		5 (18)		Beharie et al [63] Patel et al [77] Hser and Mooney [70] Wang et al [85] Hser et al [71]	
		Technology provided	Available resources		5 (18)		Griffin et al [68] Wang et al [85] Hser et al [71] Crowley and Delargy [67] Leo et al [75]	
		Clinical setting	Structural characteristics		4 (14)		Lin et al [62] Patel et al [77] Jones et al [74] Riedel et al [78]	
		Mobile medication delivery	Available resources		1 (4)		Leo et al [75]	
	**Challenges**							
		Clinical setting	Structural characteristics		4 (14)		Lin et al [62] Patel et al [77] Jones et al [74] Riedel et al [78]	
		Lack of guidelines and resources	Available resources		4 (14)		Jones et al [74] Wang et al [85] Hser et al [71] Snell-Rood et al [81]	
		Need for more administrative support	Structural characteristics		3 (11)		Calandra et al [65] Hser et al [71] Snell-Rood et al [81]	
		Cost	Cost		1 (4)		O’Gurek [76]	
		Slow uptake of virtual primary care	Capability		3 (11)		Jones et al [74] Hser et al [71] Snell-Rood et al [81]	
**Individual: innovation deliverer (clinicians)**								
	**Benefits**							
		Patient stability	Opportunity		5 (18)		Sivakumar et al [80] Calandra et al [65] Riedel et al [78] Caton et al [66] Wilson et al [86]	
		Higher patient attendance	Need		5 (18)		Uscher-Pines et al [84] Calandra et al [65] O’Gurek [76] Hser et al [71] Caton et al [66]	
		Ability to provide care virtually	Capability		3 (11)		Uscher-Pines et al [84] Huskamp et al [72] Riedel et al [78]	
		Experience with OAT^b^ or virtual primary care	Capability		3 (11)		Huskamp et al [72] Jones et al [74] Riedel et al [78]	
		Continuity of care	Need		3 (11)		Calandra et al [65] Hser et al [71] Leo et al [75]	
		Desire for virtual care	Motivation		2 (7)		Uscher-Pines et al [84] Riedel et al [78]	
		Patient bias	Need		1 (4)		Wang et al [85]	
		Convenience and efficiency	Need		1 (4)		O’Gurek [76]	
		Video appointments for higher-risk patients	Need		1 (4)		Riedel et al [78]	
		Younger clinician age	Capability		1 (4)		Jones et al [74]	
		Managing clinical complexity	Need		1 (4)		Hser and Mooney [70]	
	**Challenges**							
		Higher risk and liability	Need		10 (36)		Lin et al [62] Sivakumar et al [80] Uscher-Pines et al [84] Huskamp et al [72] Jones et al [74] Wang et al [85] Riedel et al [78] Caton et al [66] Wilson et al [86] Crowley and Delargy [67]	
		Hinders patient relations	Need		3 (11)		Beharie et al [63] Uscher-Pines et al [84] Hser et al [71]	
		Phone insufficient for care needed	Need		3 (11)		Sivakumar et al [80] Huskamp et al [72] Riedel et al [78]	
		Clinician preference for in-person care	Motivation		3 (11)		Huskamp et al [72] Jones et al [74] Riedel et al [78]	
		Limited support or guidelines available	Need		3 (11)		Wang et al [85] Hser et al [71] Snell-Rood et al [81]	
		Lack of technology knowledge or equipment	Capability		3 (11)		Uscher-Pines et al [84] Jones et al [74] Riedel et al [78]	
		Patient needing in-person care	Need		2 (7)		Uscher-Pines et al [84] Calandra et al [65]	
		Future of virtual primary care unclear	Motivation		2 (7)		Uscher-Pines et al [84] Hodgkin et al [69]	
		Shorter appointment times	Opportunity		1 (4)		Uscher-Pines et al [84]	
		Male clinician	Other		1 (4)		Jones et al [74]	
**Individual: innovation recipient (patients)**								
	**Benefits**							
		Care access	Need		13 (46)		Lin et al [62] Sivakumar et al [80] Uscher-Pines et al [84] Calandra et al [65] Hser and Mooney [70] O’Gurek [76] Wang et al [85] Hser et al [71] Sahu et al [79] Riedel et al [78] Caton et al [66] Crowley and Delargy [67] Leo et al [75]	
		Travel and transportation limitations	Need		10 (36)		Akoto [61] Uscher-Pines et al [84] Calandra et al [65] Hser and Mooney [70] O’Gurek [76] Wang et al [85] Sahu et al [79] Riedel et al [78] Caton et al [66] Crowley and Delargy [67]	
		Rurality	Need		6 (21)		Lin et al [62] Calandra et al [65] Hser and Mooney [70] Hser et al [71] Crowley and Delargy [67] Taylor et al [82]	
		Concerns about stigma	Need		4 (14)		Sivakumar et al [80] Incze et al [73] Hser and Mooney [70] Wang et al [85]	
		Flexibility of appointments	Need		4 (14)		Incze et al [73] Calandra et al [65] Wang et al [85] Sahu et al [79]	
		Continuity of care	Need		3 (11)		Lin et al [62] Sahu et al [79] Caton et al [66]	
		Convenience and efficiency	Need		2 (7)		Sivakumar et al [80] Uscher-Pines et al [84]	
		Patient preference	Need		1 (4)		Hser and Mooney [70]	
		Younger patient age	Capability		1 (4)		Lin et al [62]	
		White ethnicity	Other		1 (4)		Lin et al [62]	
		Female sex	Other		1 (4)		Lin et al [62]	
	**Challenges**							
		Digital divide	Capability		12 (43)		Griffin et al [68] Sivakumar et al [80] Patel et al [77] Uscher-Pines et al [84] Huskamp et al [72] Calandra et al [65] Hser and Mooney [70] O’Gurek [76] Wang et al [85] Hser et al [71] Snell-Rood et al [81] Wilson et al [86]	
		Mistrust of virtual care	Motivation		3 (11)		Huskamp et al [72] Hser and Mooney [70] Hser et al [71]	
		Black ethnicity	Other		2 (7)		Lin et al [62] Patel et al [77]	
		Lack of private and secure spaces	Need		2 (7)		Jones et al [74] Hser et al [71]	
		Older patient age	Capability		1 (4)		Wang et al [85]	
		Minority ethnicity	Other		1 (4)		Wang et al [85]	
		Hinders patient relations	Need		1 (4)		Beharie et al [63]	
		Cost	Cost		1 (4)		Jones et al [74]	
		Virtual care not aligned with treatment philosophy	Need		1 (4)		Hser et al [71]	
		Longer wait for appointment	Need		1 (4)		Hser et al [71]	
		English as a second language	Capability		1 (4)		Wang et al [85]	

^a^No corresponding data.

^b^OAT: opioid agonist therapy.

### Innovation: Virtual Care

Primary care (including but not limited to OAT initiation and management) was often delivered through telephone appointments (18/28, 64%), with fewer papers focusing on video modalities (14/28, 50%) or not specifying the type of synchronous virtual visits (9/28, 32%; Table 4). Some studies (7/28, 25%) emphasized the importance of providing multiple modality options, such as virtual and in-person appointments (ie, hybrid).

**Table 4:** Primary care setting, care provided, and care modality in the included studies.

Study	Primary care setting	Care provided	Care modality
Akoto [61]	Primary care clinic and outpatient treatment	OAT^a^ follow-up	Video and in person
Beharie et al [63]	Primary care clinic and outpatient treatment	OAT initiation, OAT follow-up, and other	Phone and in person
Behrends et al [64]	Community health; syringe program	Nonspecific OAT, mental health, harm reduction, and primary care	Nonspecific telemedicine^b^
Calandra et al [65]	Community health and academic training center	Nonspecific OAT, mental health, and STBBI^c^	Video, phone, and in person
Caton et al [66]	Primary care clinic, rural, and community health	OAT initiation, OAT follow-up, and mental health	Video, phone, and in person
Crowley and Delargy [67]	Addiction management in primary care program	OAT initiation, OAT follow-up, and mental health	Video, phone, and in person
Griffin et al [68]	Outreach	OAT initiation, OAT follow-up, mental health, STBBI, and primary care	Phone
Hedden et al [36]	Not specified	Nonspecific OAT, mental health, STBBI, and other	Video and phone
Hodgkin et al [69]	Primary care clinic, community health, and federally qualified health centers	Nonspecific OAT	Nonspecific telemedicine
Hser and Mooney [70]	Primary care clinic and rural	Nonspecific OAT	Video and phone
Hser et al [71]	Primary care clinic and rural	Nonspecific OAT and mental health	Video, phone, and in person
Huskamp et al [72]	Primary care clinic, rural, and outpatient treatment	Nonspecific OAT and OAT initiation	Phone and in person
Incze et al [73]	Primary care clinic	Nonspecific OAT, STBBI, and primary care	Nonspecific telemedicine and in person
Jones et al [74]	Primary care clinic, rural, outpatient treatment, community health, veteran affairs, urgent care, and long-term care facilities	OAT initiation, OAT follow-up, mental health, and harm reduction	Video and phone
Lee et al [60]	Primary care clinic	OAT initiation, mental health, STBBI, and primary care	Phone and in person
Leo et al [75]	Primary care clinic, community health, and outreach	Nonspecific OAT and OAT initiation	Nonspecific telemedicine
Lin et al [62]	Veteran affairs	Nonspecific OAT, mental health, primary care, and other	Nonspecific telemedicine and in person
O’Gurek [76]	Primary care clinic and outpatient treatment	Nonspecific OAT, mental health, and primary care	Video, phone, and in person
Patel et al [77]	Not specified	OAT initiation	Nonspecific telemedicine
Riedel et al [78]	Primary care clinic	Nonspecific OAT	Video, phone, and in person
Sahu et al [79]	Primary care clinic	Nonspecific OAT	Nonspecific telemedicine and in person
Sivakumar et al [80]	Outreach and syringe program	OAT initiation, OAT follow-up, mental health, harm reduction, and STBBI	Video, phone, and in person
Snell-Rood et al [81]	Primary care clinic and rural	Nonspecific OAT and mental health	Nonspecific telemedicine
Taylor et al [82]	Community health	Nonspecific OAT	Phone
Thompson et al [83]	Primary care clinic and rural	Nonspecific OAT	Nonspecific telemedicine and in person
Uscher-Pines et al [84]	Primary care clinic and community health	OAT initiation and OAT follow-up	Video, phone, and in person
Wang et al [85]	Primary care clinic and rural	OAT initiation	Video and phone
Wilson et al [86]	Primary care clinic, rural, and residency programs	OAT initiation, OAT follow-up, and mental health	Phone and in person

^a^OAT: opioid agonist therapy.

^b^Studies that did not explicitly state the use of synchronous telemedicine for opioid use disorder care were assumed to use this method. This assumption was based on the waiver stipulations of the Ryan Haight Act and the associated Substance Abuse and Mental Health Services Administration guidelines, which mandate the use of “real-time” (ie, audio or visual) modalities for the provision of buprenorphine [87].

^c^STBBI: sexually transmitted and blood-borne infections.

Where studies described primary care prescription of OAT, most (16/28, 57%) referred to provision generally without specific mention of the stage or length of care. Others described OAT initiation (13/28, 46%) or follow-up and stabilization (9/28, 32%) for management of OUD virtually. Outside of providing OAT, mental health care was most commonly provided (14/28, 50%), followed by treatments for sexually transmitted and blood-borne infections (6/28, 21%) [68]. In addition, harm reduction services were provided (such as syringe exchange and naloxone distribution with the support of phone consultations and home deliveries [80]), although to a lesser extent (3/28, 11%). Few studies (6/28, 21%) described other types of primary care provided (eg, on-site vaccinations [64] and in-person diabetes management [73]) and lacked specific details on the care provided (eg, it was listed as “other health needs” [63] or “other clinics” [62]).

### Outer Setting: State

The most common facilitator described in the included studies was regulatory changes (11/28, 39%) to support the delivery of virtual primary care for OUD, such as the waiver of the Ryan Haight Act in the United States, which allowed clinicians to prescribe buprenorphine without the previously required first in-person appointment [77]. Another common facilitator was COVID-19 public health measures (4/28, 14%). Due to the increased COVID-19 risk to patients and clinicians associated with in-person appointments, people with OUD could use virtual primary care to adhere to social distancing measures and reduce their COVID-19 exposure risk [84]. The reduction of COVID-19 exposure risk was also described as a benefit of virtual care.

Regulatory barriers were less commonly described (7/28, 25%) but included uncertainty in the permanence of the COVID-19 regulatory changes [72] and limited flexibility in providing methadone via virtual modalities [80]. Billing structures were described as both facilitators and challenges. For example, new billing structures or fee codes introduced during the COVID-19 pandemic (5/28, 18%) facilitated virtual care delivery. However, some clinicians experienced challenges with billing (5/28, 18%), stating that payment models did not equally compensate virtual and in-person care [65] or that virtual engagement was not included as a billable service [63]. Insurance policies yielded negative perspectives (4/28, 14%), where inconsistent [71] or insufficient [69,78] coverage of virtual care and challenges with reimbursement [86] were reported. Urine drug screenings were mentioned in 39% (11/28) of the articles [60,62,63,66,70-72,74,80,85,86]. Some articles described that changes to federal mandates during the COVID-19 pandemic reduced (3/28, 11%) [66,80,86] and facilitated innovative approaches to urine drug screens (3/28, 11%) [70-72], such as take-home kits or remote viewing.

### Inner Setting: Primary Care

Several studies (6/28, 21%) described clinical resources or guidelines as essential factors for facilitating virtual primary care implementation, ensuring that clinicians and patients could navigate virtual appointments. Such guidelines ranged from documents produced by the Substance Abuse and Mental Health Services Administration to guide clinics through policy changes to providing OAT virtually [70] or individual clinic protocols for virtual delivery of OUD care [76]. Other resources included the provision of communication devices to clinicians and patients (5/28, 18%) and training or education for patients to prepare for their virtual appointments (5/28, 18%). A total of 14% (4/28) of the studies noted that certain primary care clinics were better positioned to adopt virtual primary care. For example, clinics in urban or community-based settings [74] or larger clinics [77] were more likely to offer virtual OAT induction, although the reasoning behind these differences was not explained in the research.

Challenges affecting implementation at the clinic level included the need for more administrative support for physicians (4/28, 14%) and a lack of guidelines and resources (4/28, 14%) to support clinicians in providing evidence-based care (eg, lack of protocols for virtual primary care [85]). The studies also highlighted the challenges associated with specific clinical settings (4/28, 14%), noting that accessing OUD care without an in-person examination was more difficult in primary care solo practices versus specialty substance use facilities, opioid treatment programs, community clinics, and federally managed health administrations [74]. Furthermore, primary care practices in rural settings were less likely to provide virtual primary care services, possibly due to the lower demand for virtual services as COVID-19 risk was lower than in urban areas [74]. A few studies (3/28, 11%) described low demand for virtual care in general from both patients and clinicians [71,74,81], resulting in slower uptake in some clinics.

### Individual Characteristics (Recipient): People With OUD

The most common benefit for patients was the high accessibility of virtual care (13/28, 46%), particularly for rural patients (6/28, 21%). This advantage was linked to other benefits, such as alleviating travel challenges (10/28, 36%) and flexibility of appointments (4/28, 14%). Factors such as confidence in virtual care (2/28, 7%), its perceived convenience and efficiency (2/28, 7%), improved continuity of care (3/28, 11%), and the ability of virtual modalities to address patients’ stigma concerns (4/28, 14%) also emerged as pivotal factors. In addition, younger age (1/28, 4%) and cost savings (2/28, 7%) positively influenced patients’ engagement with virtual primary care.

The gap between people who can easily use and access technology and those who cannot (ie, “the digital divide”) remains the primary obstacle to the uptake of virtual care for people with OUD (12/28, 43%). This is compounded by a lack of technological knowledge or equipment (6/28, 21%), mistrust of virtual care (3/28, 11%), and absence of a private or secure space for appointments (2/28, 7%). Intersectionality between specific marginalizing patient attributes, such as being an older adult (1/28, 4%) and racialization (3/28, 11%), resulted in inequity in virtual primary care access due to structural barriers of the health care system.

### Individual Characteristics (Provider): Clinicians

Clinicians found virtual care beneficial, particularly when managing stable patients (5/28, 18%) and patients who could attend appointments consistently (5/28, 18%). Clinicians also found virtual care beneficial for continuity of care (3/28, 11%). Furthermore, previous experience with OAT or using virtual modalities (3/28, 11%) and a genuine interest in delivering virtual primary care (2/28, 7%) facilitated its adoption among clinicians.

Conversely, apprehension about liability or heightened risks associated with offering virtual primary care for people with OUD (10/28, 36%) were seen as challenges among clinicians. Concerns also emerged over the adequacy of phone-based relative to video-based appointments (3/28, 11%), challenges of fostering a therapeutic relationship through virtual visits (3/28, 11%), and the belief that in-person care was necessary for patients (2/28, 7%). Some clinicians also reported a preference for in-person care (3/28, 11%). Additional barriers included technological challenges (3/28, 11%) such as low digital literacy and inadequate technology equipment.

### Quality Appraisal

Over a quarter of the studies (8/28, 29%), most of which (7/8, 88%) were original research, received high scores through the QuADS tool. In contrast, commentaries (5/28, 18%) and conference abstracts (5/28, 18%) predominantly received lower scores. The scores obtained through our quality assessment using the QuADS tool are grouped by study type (Table 5).

**Table 5:** Quality appraisal of the included studies grouped by study type and Quality Assessment With Diverse Studies score [55].

	Low quality (score of 0-12)	Medium quality (score of 13-25)	High quality (score of 26-39)
Original research	—^a^	Lee et al [60] Sivakumar et al [80] Uscher-Pines et al [84] Snell-Rood et al [81]	Lin et al [62] Beharie et al [63] Behrends et al [64] Huskamp et al [72] Jones et al [74] Riedel et al [78] Caton et al [66]
Conference abstract	Griffin et al [68] Incze et al [73] Sahu et al [79] Taylor et al [82] Thompson et al [83]	Akoto [61]	—
Commentary	Calandra et al [65] Hser and Mooney [70] Wang et al [85] Wilson et al [86] Leo et al [75]	Hser et al [71]	—
Brief report	Crowley and Delargy [67]	Patel et al [77] O’Gurek [76]	—
Protocol	—	—	Hedden et al [36]
Review	Hodgkin et al [69]	—	—

^a^No corresponding data.

## Discussion

### Principal Findings

In reviewing 28 studies on the experiences of people with OUD and primary care clinicians with virtual modalities in primary care, we sought to explore the current state of the literature, identify research gaps, and provide insights into the potential benefits and challenges of virtual approaches. The CFIR constructs were instrumental in our investigation, guiding us in identifying the organizational-, structural-, and individual-level factors that influence the implementation and ongoing use of virtual primary care for people with OUD. Our findings underscore that, with careful consideration of the implementation factors at all levels, virtual modalities in primary care hold promise as a feasible, acceptable, and effective option for people with OUD as complements to in-person primary care, aligning with research in other settings [88-90].

Our review highlights that research related to virtual primary care for the management of OUD is limited and even more so for the broad range of primary care provided for people with OUD beyond OAT despite the rapid increase in the availability of virtual care resulting from the COVID-19 pandemic. The available details on the range of primary care services offered virtually were scarce, but they did include areas such as mental health care, sexual health care, and harm reduction services. For example, people with OUD are known to frequently experience comorbid conditions (eg, HIV/AIDS, hepatitis C, and hepatitis B [91]), infections (eg, endocarditis and septic arthritis [92]), and other frequent or episodic health care needs (eg, wound care [93]) that require timely and ongoing primary care. However, the extant literature on virtual primary care for people with OUD focuses on management of OUD and not on other primary care needs, and few of the included studies (6/28, 21%) reported how or whether these conditions were addressed. This remains a core knowledge gap in the literature.

Relying on virtual modalities to increase access to health care was a common finding [94]. Virtual visits remove the necessity for physical travel to medical appointments and reduce associated costs (eg, transit or gas, taking time off work, and childcare). By offering the convenience of accessing care from a smartphone or other communication devices, virtual care can also support continuous access to and retention in OAT [95]. Patient lifestyle and stability play a significant role in driving their use of virtual care. Individuals with childcare responsibilities [79,85], limited flexibility during work hours [65,79], or those who are more stable on OAT [65,78,86] may find virtual care well-suited to meeting their care needs, while minimizing disruptions to their day-to-day lives.

The digital divide, the gap between those who can use and access technology easily and those who cannot, emerged as a central theme in multiple studies (12/28, 43%). Disparities in the components of the digital divide—such as access to technology, internet connectivity, and digital literacy—can affect the ability of people with OUD to use virtual care and its effectiveness. Furthermore, clinicians’ belief that virtual primary care was unsuitable for some patients, too risky due to patients’ health status, or associated with high liability also hindered its adoption, aligning with concerns found in other (ie, non–primary care) clinical settings [96,97]. With the challenges associated with patient stability and social determinants of health (eg, digital technology access, longevity of OAT treatment, and housing), providing care to people with OUD using solely virtual modalities may unintentionally limit equitable health care access. However, clinicians who believe that virtual primary care is unsuitable for unstable patients may be ignoring patient preferences [70]. While virtual primary care for less stable patients is a nuanced topic and needs concerted research efforts and practice approaches, evidence is emerging that virtual primary care can provide a safe supplement to in-person visits for unstable or new patients [72,78,98]. To enhance the effectiveness of virtual primary care for this population, offering diverse appointment types can accommodate patient preferences, socioeconomic factors, and health circumstances, fostering patient autonomy and promoting patient-centered care.

Virtual primary care for people with OUD is promising and feasible. However, this review also highlights that there may be disparities based on patient age [62,85]; language [85]; race or ethnicity [62,77,85]; sex [62]; and experience of the digital divide [65,68,70-72,76,77,80,81,84-86] due to rurality, socioeconomic status, or limited access to technology. These disparities pose challenges for implementing and accessing virtual care [99]. Without deliberate efforts to address these access barriers, virtual primary care could perpetuate inequities and worsen existing disparities in OAT access [100-102].

### Limitations

There are several limitations to this scoping review. Scoping reviews inherently possess limitations in terms of depth of analysis and generalizability [39], and the possibility of missing studies needs to be acknowledged. This is attributable to multiple factors; among them is our stringent inclusion criteria, which not only resulted in excluding studies with unclear or inadequately described settings but also affected our interrater reliability score. Furthermore, our definition of virtual visits excluded asynchronous forms, and we did not consider care settings that provided only OAT, which excludes the recent growth of literature on digital services for treatment of OUD as they take place within stand-alone addiction treatment services. This criterion was in place to align with our broader research project [36] and maintain a manageable review size. We acknowledge that there are valuable lessons from virtual care models provided for other populations and conditions, which may enrich our understanding and approach in this area but were not captured in this review. In addition, studies published in languages other than English were not captured. There was also a variance in the quality of the included studies. Some of the included studies (12/28, 43%) received low QuADS scores due to limitations in study information (eg, conference abstracts with restricted word counts). Furthermore, despite the growing demand for research that distinguishes between experiences associated with sex and gender [103,104], many of the studies we reviewed appear to conflate these distinct experiences (eg, reporting participant gender as female or male) or did not report any sex- or gender-based analyses. In addition, across the studies we reviewed, the heterogeneous research methods complicated data comparison and aggregation. Most studies (25/28, 89%) were conducted in the United States, which can limit or challenge generalizability to other health system contexts. For example, regulations and billing, part of the CFIR’s outer setting domain, differ dramatically across settings [105-107]. While qualified physicians in the United States and Canada can prescribe buprenorphine, the United States previously required a waiver under the Drug Addiction Treatment Act, whereas there is no such requirement in Canada [108]. Similarly, methadone treatment requires access to specialized clinics (ie, opioid treatment programs) in the United States [109], but in Canada, it can be prescribed in various health care settings [110]. Nonetheless, this scoping review presents novel results as the first of its kind covering virtual primary care for people with OUD. Our methodologically rigorous approach and the quality appraisal of the included studies make this review a valuable contribution to the literature given the sparse research in this field.

### Conclusions

This scoping review highlights the promise of virtual primary care as a feasible, acceptable, and effective option for people with OUD, particularly in enhancing accessibility and continuous access to care. However, addressing the digital divide and mitigating disparities based on demographic and socioeconomic factors will be critical in realizing the full potential of virtual care. Future research and policy initiatives should prioritize efforts to bridge these gaps, ensuring that virtual primary care becomes an inclusive and equitable platform for delivering comprehensive care to and for people with OUD. In addition, there is a need for future research to explore the use of virtual modalities for primary care needs beyond OAT management to fully understand and enhance virtual care for people with OUD.

## Appendix

Multimedia Appendix 1 PRISMA-ScR (Preferred Reporting Items for Systematic Reviews and Meta-Analyses extension for Scoping Reviews) checklist.

Multimedia Appendix 2 Final search strategy.

Multimedia Appendix 3 Data extraction template.

Multimedia Appendix 4 Quality assessment template.

## Abbreviations

CFIR: Consolidated Framework for Implementation Research
OAT: opioid agonist therapy
OUD: opioid use disorder
PRISMA: Preferred Reporting Items for Systematic Reviews and Meta-Analyses
PRISMA-ScR: Preferred Reporting Items for Systematic Reviews and Meta-Analyses extension for Scoping Reviews
QuADS: Quality Assessment With Diverse Studies

## Data Availability

All data generated or analyzed during this study are included in this published article and its supplementary information files.
