# Supplementary material for: Virtual Primary Care for People With Opioid Use Disorder: Scoping Review of Current Strategies, Benefits, and Challenges
Source: J Med Internet Res. 2024 Dec 2;26:e54015. doi: 10.2196/54015 (PMC11650090; doi:10.2196/54015)
Supplement: Multimedia Appendix 2 [file jmir_v26i1e54015_app2.pdf]

Appendix II: Final search strategy

MEDLINE (Ovid): 263 hits

Ovid MEDLINE(R) and Epub Ahead of Print, In-Process, In-Data-Review & Other Non-Indexed Citations, Daily and Versions <1946 to December 06, 2022>

1	exp Analgesics, Opioid/ or exp Narcotics/ or Opiate Substitution Treatment/ or exp Narcotic-Related Disorders/ or exp Drug Overdose/	162063
2	(opioid* or opiate* or narcotic* or PWUO or PWOUD or Alphaprodine or Buprenorphine or Butorphanol or Codeine or Dextromoramide or Dextropropoxyphene or Dihydromorphine or Diphenoxylate or Enkephalin or Ethylketocyclazocine or Ethylmorphine or Etorphine or Fentanyl or Heroin or Hydrocodone or Hydromorphone or Levorphanol or Meperidine or Meptazinol or Methadone or Methadyl Acetate or Morphine or Nalbuphine or Opium or Oxycodone or Oxymorphone or Pentazocine or Phenazocine or Phenoperidine or Pirinitramide or Promedol or Remifentanil or Sufentanil or Tapentadol or Tilidine or Tramadol).mp.	275392
3	((addict* or depend* or "use" or user* or usage) adj3 (opioid* or opiate* or narcotic*)).mp.	35856
4	1 or 2 or 3	283930
5	exp Telemedicine/ or exp Telecommunications/ or internet-based intervention/ or computers, handheld/ or smartphone/ or Patient Portals/	125017
6	("video call" or telephon* or phone* or "online clinic" or "virtual clinic" or "virtual health" or "online health" or e-health or e?health or m-health or m?health or digital-health or tele?health or tele-health or tele-monitor* or tele?monitor* or tele?communication* or tele-communication* or synchronous or skype or zoom or tele-medicine or tele?medicine or smart-device* or smart?device* or smart-phone or "smart?phone" or "cell-phone" or "mobile device" or "mobile phone" or "iPhone" or "mobile health" or "e?care" or e-care or e?consult* or e?counsel* or e-consult*or e?diagnos* or e-diagnos* or e?medicine or e-medicine or e?nurs* or e-nurs* or e?physician or e-physician or e-doctor* or e?doctor* or e?referral or e-referral or e?treatment* or e-treatment* or e?clinic* or e-clinic* or e?appointment* or e-appointment* or e?monitor* or e-monitor* or tele-care or telenurs* or tele-nurs* or "remote medicine" or "remote health" or "distance medicine" or "digital health" or "patient web portal*" or "patient web-portal*" or "patient portal*" or "web portal*").mp.	270138
7	5 or 6	321191
8	nurse practitioners/ or nurses, community health/ or nurses, public health/ or general practitioners/ or physicians, family/ or physicians, primary care/ or community health services/ or primary health care/ or exp general practice/ or clinical medicine/ or community medicine/	233729
9	("primary care" or "primary care physician*" or "general practic*" or "general practitioner*" or "nurse practitioner*" or "family practic*" or "family doctor" or "clinician*").mp.	556988
10	8 or 9	626235
11	4 and 7 and 10	318
12	limit 11 to (english language and humans)	263

Appendix II: Final search strategy

EMBASE (Ovid): 780 hits

1	exp narcotic agent/ or exp opiate/ or exp opiate addiction/ or exp methadone/ or exp opiate substitution treatment/ or exp buprenorphine/ or drug dependence treatment/ or exp methadone treatment/ or drug overdose/ or opiate overdose/	400040
2	(opioid* or opiate* or narcotic* or PWUO or PWOUD or Alphaprodine or Buprenorphine or Butorphanol or Codeine or Dextromoramide or Dextropropoxyphene or Dihydromorphine or Diphenoxylate or Enkephalin or Ethylketocyclazocine or Ethylmorphine or Etorphine or Fentanyl or Heroin or Hydrocodone or Hydromorphone or Levorphanol or Meperidine or Meptazinol or Methadone or Methadyl Acetate or Morphine or Nalbuphine or Opium or Oxycodone or Oxymorphone or Pentazocine or Phenazocine or Phenoperidine or Pirinitramide or Promedol or Remifentanil or Sufentanil or Tapentadol or Tilidine or Tramadol).mp. [mp=title, abstract, heading word, drug trade name, original title, device manufacturer, drug manufacturer, device trade name, keyword heading word, floating subheading word, candidate term word]	470023
3	exp telecommunication/ or exp telemedicine/ or exp web-based intervention/ or computer/ or exp smartphone/ or exp mobile phone/	225221
4	("video call" or telephon* or phone* or "online clinic" or "virtual clinic" or "virtual health" or "online health" or e-health or e?health or m-health or m?health or digital-health or tele?health or tele-health or tele-monitor* or tele?monitor* or tele?communication* or tele-communication* or synchronous or skype or zoom or tele-medicine or tele?medicine or smart-device* or smart?device* or smart-phone or "smart?phone" or "cell-phone" or "mobile device" or "mobile phone" or "iPhone" or "mobile health" or "e?care" or e-care or e?consult* or e?counsel* or e-consult*or e?diagnos* or e-diagnos* or e?medicine or e-medicine or e?nurs* or e-nurs* or e?physician or e-physician or e-doctor* or e?doctor* or e?referral or e-referral or e?treatment* or e-treatment* or e?clinic* or e-clinic* or e?appointment* or e-appointment* or e?monitor* or e-monitor* or tele-care or telenurs* or tele-nurs* or "remote medicine" or "remote health" or "distance medicine" or "digital health" or "patient web portal*" or "patient web-portal*" or "patient portal*" or "web portal*").mp. [mp=title, abstract, heading word, drug trade name, original title, device manufacturer, drug manufacturer, device trade name, keyword heading word, floating subheading word, candidate term word]	387460
5	nurse practitioner/ or family nurse practitioner/ or exp general practitioner/ or exp general practice/ or exp primary medical care/ or clinical medicine/ or community medicine/	320379
6	("primary care" or "primary care physician*" or "general practic*" or "general practitioner*" or "nurse practitioner*" or "clinician*" or "family practic*" or "family doctor").mp. [mp=title, abstract, heading word, drug trade name, original title, device manufacturer, drug manufacturer, device trade name, keyword heading word, floating subheading word, candidate term word]	777605
7	1 or 2	519756
8	3 or 4	477310
9	5 or 6	810982
10	7 and 8 and 9	814
11	limit 10 to (human and english language)	780

Appendix II: Final search strategy

Web of Science: 283 hits

#1

TS=(opioid* or opiate* or narcotic* or "Narcotic-Related Disorders" or PWUO or PWOUD or Alphaprodine or Buprenorphine or Butorphanol or Codeine or Dextromoramide or Dextropropoxyphene or Dihydromorphine or Diphenoxylate or Enkephalin or Ethylketocyclazocine or Ethylmorphine or Etorphine or Fentanyl or Heroin or Hydrocodone or Hydromorphone or Levorphanol or Meperidine or Meptazinol or Methadone or "Methadyl Acetate" or Morphine or Nalbuphine or Opium or Oxycodone or Oxymorphone or Pentazocine or Phenazocine or Phenoperidine or Pirinitramide or Promedol or Remifentanil or Sufentanil or Tapentadol or Tilidine or Tramadol)

#2

TS=(telemedicine or "video call" or telephon* or phone* or "online clinic" or "virtual clinic" or "virtual health" or "online health" or e-health or e$health or m-health or m$health or digital-health or tele$health or tele-health or tele-monitor* or tele$monitor* or tele$communication* or tele-communication* or synchronous or skype or zoom or tele-medicine or tele$medicine or smart-device* or smart$device* or smart-phone or "smart$phone" or "cell-phone" or "mobile device" or "mobile phone" or "iPhone" or "mobile health" or "e$care" or e-care or e$consult* or e$counsel* or e-consult*or e$diagnos* or e-diagnos* or e$medicine or e-medicine or e$nurs* or e-nurs* or e$physician or e-physician or e-doctor* or e$doctor* or e$referral or e-referral or e$treatment* or e-treatment* or e$clinic* or e-clinic* or e$appointment* or e-appointment* or e$monitor* or e-monitor* or tele-care or telenurs* or tele-nurs* or "remote medicine" or "remote health" or "distance medicine" or "digital health" or "patient web portal*" or "patient web-portal*" or "patient portal*" or "web portal*")

#3

TS=("primary care" or "primary care physician*" or "general practic*" or "general practitioner*" or "nurse practitioner*" or "family practic*" or "family doctor" or "family physician" or "community medicine" or clinician*)

Appendix II: Final search strategy

CINAHL: 142 hits

S11	S7 AND S8 AND S9 - Limiters - English Language; Human	(142)
S10	S7 AND S8 AND S9	(228)
S9	S5 OR S6	330,018)
S8	S3 OR S4	(228,801)
S7	S1 OR S2	(89,461)
S6	"primary care" or "primary care physician*" or "general practic*" or "general practitioner*" or "nurse practitioner*" or "family practic*" or "family doctor" or "clinician*"	(271,074)
S5	(MH "nurse practitioners") or (MH "nurses, community health") or (MH "nurses, public health") or (MH "general practitioners") or (MH "physicians, family") or (MH "physicians, primary care") or (MH "community health services") or (MH "primary health care") or (MH "general practice+") or (MH "clinical medicine") or (MH "community medicine")	(131,443)
S4	(MH Telemedicine+) or (MH Telecommunications+) or (MH "internet-based intervention") or (MH "computers, handheld") or (MH smartphone) or (MH Patient Portals)	(162,798)
S3	"video call" or telephon* or phone* or "online clinic" or "virtual clinic" or "virtual health" or "online health" or e-health or e#health or m-health or m#health or digital-health or tele#health or tele-health or tele-monitor* or tele#monitor* or tele#communication* or tele-communication* or synchronous or skype or zoom or tele-medicine or tele#medicine or smart-device* or smart#device* or smart-phone or "smart#phone" or "cell-phone" or "mobile device" or "mobile phone" or "iPhone" or "mobile he ...	(128,964)
S2	opioid* or opiate* or narcotic* or PWUO or PWOUD or Alphaprodine or Buprenorphine or Butorphanol or Codeine or Dextromoramide or Dextropropoxyphene or Dihydromorphine or Diphenoxylate or Enkephalin or Ethylketocyclazocine or Ethylmorphine or Etorphine or Fentanyl or Heroin or Hydrocodone or Hydromorphone or Levorphanol or Meperidine or Meptazinol or Methadone or Methadyl Acetate or Morphine or Nalbuphine or Opium or Oxycodone or Oxymorphone or Pentazocine or Phenazocine or Phenoperidine or Pirin ...	(85,738)
S1	(MH "Analgesics, Opioid+") OR (MH "Narcotics+") OR (MH "Opiate Substitution Treatment") or (MH "Narcotic-Related Disorders") or (MH "Drug Overdose+")	(58,289)

Appendix II: Final search strategy

Grey Literature Search

(opioid* OR opiate* OR narcotic* OR PWUO OR PWOUD OR Buprenorphine OR Codeine OR Fentanyl OR Heroin OR Hydrocodone OR Hydromorphone OR Methadone Morphine OR Opium OR Oxycodone OR Oxymorphone OR Sufentanil OR Tramadol) AND summary:("video call" OR telephone OR "online clinic" OR "virtual clinic" OR "virtual health" OR "online health" OR e-health OR m-health OR “digital health” OR tele-health OR skype OR zoom OR telemedicine) AND summary:("primary care" OR "primary care physician" OR "general practice" OR "general practitioner" OR "nurse practitioner" OR "family practice" OR "family doctor")
