# Supplementary material for: Virtual Primary Care for People With Opioid Use Disorder: Scoping Review of Current Strategies, Benefits, and Challenges
Source: J Med Internet Res. 2024 Dec 2;26:e54015. doi: 10.2196/54015 (PMC11650090; doi:10.2196/54015)
Supplement: Multimedia Appendix 3 [file jmir_v26i1e54015_app3.pdf]

Appendix III: Data Extraction Template

**General Information**	
Title	
Author details	
Paper type	• Original Research<br>• Brief Report<br>• Review Paper<br>• Conference Abstract<br>• Commentary<br>• Other
Country in which study was conducted	• United States<br>• UK<br>• Canada<br>• Australia<br>• Other
Publication date	
Funding	
**Characteristics of Included Studies**	
Methods	
Purpose of study	
Study design	• Randomised controlled trial<br>• Non-randomised experimental study<br>• Cohort study<br>• Cross sectional study<br>• Case control study<br>• Systematic review<br>• Qualitative research<br>• Prevalence study<br>• Case series<br>• Case report<br>• Diagnostic test accuracy study<br>• Clinical prediction rule<br>• Economic evaluation<br>• Text and opinion<br>• Other
Population description	
Total number of participants	
Description of intervention/study design	
Type of care for PWOUD	• OAT initiation<br>• OAT follow up<br>• OAT/MOUD general (not specified)<br>• Syringe services<br>• STBBI/HCV/HIV<br>• Mental health (eg. Anxiety/Depression/PTSD)<br>• Other
Setting of primary care facility	• Family physician clinic<br>• Walk in clinic<br>• Rural clinic<br>• Veterans (VA Hospital/Clinic)<br>• Outpatient OAT/SUD treatment<br>• Community health centres/clinics<br>• Outreach/mobile clinic<br>• Primary care not specified<br>• Other
Modalities	• FaceTime/Zoom Call (Video Conference)<br>• Telephone (Audio only)<br>• In-person<br>• Telemedicine not specified<br>• Other
**Results**	
Patient outcomes or results	
Facilitators	
Barriers	
Relevant health system features	• Private fee and co-pay (finance)<br>• Free/universal access (finance)<br>• Medicare/Medicaid (finance)<br>• Uninsured (finance)<br>• Team-based care/interdisciplinary<br>• Clinicians/Teams Private (delivery)<br>• Clinicians/Teams Public (delivery)<br>• Low-barrier access (if yes include in other)<br>• Other
Future research	
Notes	
